# The Association between Serum 25(OH)D Status and Blood Pressure in Participants of a Community-Based Program Taking Vitamin D Supplements

**DOI:** 10.3390/nu9111244

**Published:** 2017-11-14

**Authors:** Naghmeh Mirhosseini, Hassanali Vatanparast, Samantha M. Kimball

**Affiliations:** 1 Pure North S’Energy Foundation, Calgary, AB T2R 0C5, Canada; Naghmeh.Mirhosseini@purenorth.ca; 2 College of Pharmacy and Nutrition, University of Saskatchewan, Saskatoon, SK S7N 5C9, Canada; vatan.h@usask.ca

**Keywords:** blood pressure, vitamin D supplement, hypertension, 25-hydroxyvitamin D, medication

## Abstract

Background: Vitamin D deficiency is a risk factor for hypertension. Methods: We assessed 8155 participants in a community-based program to investigate the association between serum 25-hydroxyvitamin D (25(OH)D) status and blood pressure (BP) and the influence of vitamin D supplementation on hypertension. Participants were provided vitamin D supplements to reach a target serum 25(OH)D > 100 nmol/L. A nested case-control study was conducted to examine the effect of achieving physiological vitamin D status in those who were hypertensive and not taking BP-lowering medication, and hypertensive participants that initiated BP-lowering medication after program entry. Results: At baseline, 592 participants (7.3%) were hypertensive; of those, 71% were no longer hypertensive at follow-up (12 ± 3 months later). There was a significant negative association between BP and serum 25(OH)D level (systolic BP: coefficient = −0.07, *p* < 0.001; diastolic BP: coefficient = −0.1, *p* < 0.001). Reduced mean systolic (−18 vs. −14 mmHg) and diastolic (−12 vs. −12 mmHg) BP, pulse pressure (−5 vs. −1 mmHg) and mean arterial pressure (−14 vs. −13 mmHg) were not significantly different between hypertensive participants who did and did not take BP-lowering medication. Conclusion: Improved serum 25(OH)D concentrations in hypertensive individuals who were vitamin D insufficient were associated with improved control of systolic and diastolic BP.

## 1. Introduction

Hypertension is a common health problem, one of the leading costs to the health care system, and a significant cause of mortality and morbidity worldwide [1]. Hypertension is also one of the most common and influential risk factors of cardiovascular disease including myocardial infarction, cerebral stroke, congestive heart failure, peripheral vascular disorders and kidney disease [2]. It has been estimated that eliminating high blood pressure would reduce the occurrence of stroke by 35% and heart attacks by 18% [3,4]. To reduce the burden of hypertension, a multicomponent lifestyle intervention that includes weight loss, increased physical activity, restricted sodium and alcohol consumption, and adherence to a Dietary Approach to Stop Hypertension (DASH) diet with plenty of fruits, vegetables, and low-fat dairy items and little saturated fat is needed [5]. Moreover, improved vitamin D status has been proposed as an easily modifiable risk factor [6].

At a cellular level, vitamin D exerts antihypertensive effects through improved endothelial function [7,8,9], reduced production of pro-inflammatory cytokines [10], reduced activity of the renin-angiotensin-aldosterone system and reduced levels of parathyroid hormone [11,12,13]. Observational studies consistently find an association between lower serum 25-hydroxyvitamin D (25(OH)D) concentrations and higher blood pressure levels [9,14,15]. However, there have been conflicting results from trials investigating vitamin D supplementation as an intervention to improve blood pressure [16,17,18,19]. The varied intervention designs may explain the inconsistent results including the baseline health of the study population (e.g., hypertensive versus normotensive), the dose range of vitamin D, daily dose versus one large bolus dose, the resultant change in 25(OH)D, duration of the trial and anti-hypertensive medication use [16,18,19,20]. Most notably, several trials have investigated the effects of vitamin D in populations with mean blood pressure values in the normotensive range for which we would not expect to see a mean reduction. In support of this, a meta-analysis conducted by Witham et al. revealed a modest and significant decrease in blood pressure in studies in which mean blood pressure was elevated at baseline [19]. Similarly, Kunutsor found a reduced risk of 12% for each 25 nmol/L increment of 25(OH)D [21]. In patients with pre-existing cardiometabolic disease, vitamin D supplementation resulted in a small decrease in blood pressure [18]. The benefit of vitamin D supplementation may only be detectable when initiated in high-risk individuals, both for high blood pressure and/or insufficient vitamin D status, rather than in normotensive individuals already in an acceptable range of vitamin D status.

Around a fifth of Canadians (18%) have high blood pressure [22]. One-third of Canadians are vitamin D-deficient with a serum 25(OH)D < 50 nmol/L and do not consume enough vitamin D to meet the Recommended Daily Allowance (RDA) [23,24,25]. It has been hypothesized that physiological levels of vitamin D, serum 25(OH)D > 100 nmol/L [26], may be beneficial for health outcomes beyond bone health and include improved blood pressure control. We analyzed data collected as part of a community-based program that focuses on optimizing health to prevent chronic disease and involves vitamin D supplementation aimed at achieving serum 25(OH)D concentrations >100 nmol/L to test this hypothesis. The objective of the current study was to characterize the association of a wide range of serum 25(OH)D levels on blood pressure in a Northern population (49–59° N) at baseline and following one year of vitamin D supplementation. Further, we investigated the effect of vitamin D supplementation in those who were hypertensive at time of entry to the program using a nested case-control design.

## 2. Materials and Methods

### 2.1. Study Design

This study is a retrospective database analysis. A dataset was constructed containing de-identified data for all participants (*n* = 8155) enrolled in the Pure North Community Program between 2010 and 2017 that met the criteria. All participants were included that had consented to the secondary use of their anonymized data for research and who had measures of blood pressure (BP) and 25(OH)D at the entry to the program (baseline) and at the follow-up (6–18 months later). Other measures were included if available including: age, sex, ethnicity, waist circumference, body mass index (BMI), vitamin B12, omega 6:3 ratio (arachidonic acid: AA, eicosapentaenoic acid: EPA), hs-CRP (high sensitivity C-reactive protein) (as a marker of inflammation), season of observation, lifestyle-related parameters (fruit and vegetable consumption, physical activity, tobacco use) and the consumption of blood pressure (BP) lowering medications. We investigated the association between vitamin D status, as well as other nutrients, and blood pressure measures and the influence of vitamin D supplementation on hypertension.

Next, we used a nested case-control design to examine the effect of vitamin D supplementation on established hypertension and pre-hypertension (at risk for established hypertension) in individuals not taking any BP-lowering medication. In case-control study 1, cases (*n* = 40) and controls (*n* = 80) were hypertensive individuals (systolic BP ≥140 mmHg and diastolic BP ≥90 mmHg) and, in case-control study 2, cases (*n* = 187) and controls (*n* = 374) were pre-hypertensive individuals (systolic BP 121-<140 mmHg and diastolic BP 81-<90 mmHg) [27]. The cases differed from controls in that they were not taking any BP-lowering medications whereas controls were and had initiated those medications after joining the Pure North program. All cases that had baseline and follow-up measures for blood pressure and serum 25(OH)D concentration were included. Controls were matched based on age, sex and BMI. Participants were excluded if they had a history of renal disorders, severe cardiovascular disease, or any severe digestive, hepatic or endocrine disorder.

### 2.2. The Pure North Community-Based Program (Intervention)

Pure North S’Energy Foundation is a not-for-profit wellness program based in Calgary, Alberta, Canada, that focuses on the prevention of chronic disease. The Pure North program offers lifestyle advice, education and nutritional supplements to its participants. There are no inclusion/exclusion criteria for entering the Pure North program and the program does not substitute for conventional health care. In addition to vitamin D supplements, the intervention program also provides other nutritional supplements as well as consultations on lifestyle modification, including diet, physical activity and help with tobacco cessation. This information is recorded for each participant which we controlled for when examining the relationship between vitamin D status and supplementation effects on hypertension.

The core tenant of the program is to achieve optimal nutritional status with a focus on physiological levels of vitamin D [26]. All participants are encouraged to achieve a 25(OH)D level above 100 nmol/L (<250 nmol/L) and because of inter-individual response differences vitamin D3 dosages are adjusted accordingly for the individual. Vitamin D3 doses are often above the upper level of intake, 4000 IU/day, to achieve the target 25(OH)D level. Vitamin D3 intake, adjusted individually under medical supervision, ranged from 1000 to 20,000 IU/day.

The program supplements were provided to everyone in individual daily packets, Vitality Packs, containing a multivitamin and multimineral formula (Vital 2 Platinum), omega-3 fatty acids (400 mg EPA and 200 mg DHA), vitamin C (1000 mg), vitamin B12 (5000 mg methylcobalamin), probiotics (*Biffidobacterium* and *Lactobacillus* strains) and vitamin D3 drops (1000 IU/drop). These supplements provide a background of optimal nutrient levels from which to assess the effect of vitamin D3 on blood pressure outcomes.

### 2.3. Program Measurements Included in the Dataset

Participants were interviewed and assessed by health care professionals at each program visit to collect demographic information, medical history, and medication use (including blood pressure lowering medications). Visits occur every 6–12 months. Body measurements included weight, in light clothing (to nearest 0.1 kg), and height (to nearest 0.5 cm), measured twice. BMI was calculated from the average weight (kg) divided by the average height (m) measurement squared. Systolic and diastolic blood pressure were measured by a trained nurse using a mercury sphygmomanometer (Welch Allyn, Hechingen, Germany), with an accuracy of 2 mmHg, with an appropriate cuff (Adult 11, 25–34 cm) on the dominant arm in the sitting position in a quiet room after a five minute rest. Three blood pressure measurements were taken, each one minute apart. To account for any white coat effect, blood pressure was measured after a five minutes rest and repeated three times. The average of three readings was used for these analyses.

Blood sample measurements collected at entry to the program (baseline) and at follow-up (range 6–18 months) were included in the dataset. Blood samples were analyzed by Doctor’s Data (St. Charles, IL, USA). Serum 25(OH)D concentrations were assayed using Liquid Chromatography-tandem Mass Spectrometry (LC-MS/MS), with an assay %CV of 2.4%. Serum vitamin B12 was measured on an automated analyzer with a chemiluminescent immunoassay (Beckman Coulter) with a CV of 7.7%. Red blood cell fatty acids were measured by gas chromatography and the ratio of arachidonic acid (AA, an omega-6 fatty acid) to eicosapentaenoic acid (EPA, an omega-3 fatty acid) calculated (AA:EPA). Pulse pressure (PP) was calculated from deducting diastolic blood pressure from systolic blood pressure. Further, Mean Arterial Pressure (MAP), an indicator of perfusion rather than systolic blood pressure, was calculated using the following formula: [1/3 (systolic BP − diastolic BP) + diastolic BP] [28].

When investigating nutrients effects, it is important that co-nutrient status is optimized in order to ensure that the tested nutrient is the only nutrition-related limiting factor in the response as outlined by Heaney [29]. The Pure North database offers an opportunity to characterize the effects of serum 25(OH)D concentrations on blood pressure measures in a population with similar intakes of other nutrients. In addition to 25(OH)D, serum vitamin B12 and omega 6:3 ratio (AA:EPA) measurements were incorporated into the analyses when available to investigate whether any difference found may be due to vitamin D3 or to optimized nutrient status in general. 

This study was approved by the Research Ethics Board at St. Mary’s University, Calgary, AB, Canada (File # 072FA2017). All participants provided written, informed consent to permit the secondary use of their data for research.

### 2.4. Statistical Analysis

Data were analyzed using SPSS (version 23; SPSS IBM, New York, NY, USA). To assess the normality of data distribution, the Kolmogorov–Smirnov test and histogram with normal curve were used. If the variables followed a normal distribution, Independent Samples T-test or ANOVA were used. Otherwise, the Mann–Whitney U-test or Kruskal–Wallis test were used to compare groups. To compare changes within groups over time, Paired-Samples T test or Wilcoxon Signed Rank test were used according to the normality of data. Binary logistic regression was used to determine the independent predictors for blood pressure change and, more specifically, the association between hypertension with serum 25(OH)D status. Regression models were corrected for confounding parameters including age, sex, ethnicity, BMI status, the season of observation, AA:EPA ratio, serum vitamin B12 status, inflammation, fruit and vegetable consumption, physical activity and tobacco use.

## 3. Results

### 3.1. Baseline Measurements

We assessed data from 8155 participants enrolled in the program between 2010 and 2017 with follow-up an average of one year later (12 ± 3 months). Baseline demographics are presented in Table 1. Among the participants, 60% were women, and the mean age was 56 ± 15 years. At baseline, 36.4% and 26.4% of participants were overweight and obese, respectively. Systolic and diastolic blood pressure and MAP were significantly lower among those participants who had higher levels of serum 25(OH)D at baseline. A significant trend was found across serum 25(OH)D concentration ranges such that as 25(OH)D increased, systolic and diastolic blood pressure decreased. Half of participants had their baseline visit in the summer (53%) and another half in the winter (47%). Of those who had their baseline visit in winter, 70% had their follow-up visit was in winter as well. Vitamin D deficiency (serum 25(OH)D <50 nmol/L) [30] was more common among those who had their baseline visit in winter months (14%) in comparison with summer months (8%). Eleven percent of participants had serum 25(OH)D concentrations below 50 nmol/L at baseline, considered deficient, and 42% had 25(OH)D levels <75 nmol/L (insufficient) [31,32]. More than 50% of participants reported not taking vitamin D or a vitamin D intake below 1000 IU/day at baseline. Vitamin D deficiency (<50 nmol/L) was more common in North American (61%) followed by Aboriginal (14%) and Asian (14%). Of those with winter visits 8.1% had hypertension at baseline and 6.2% at one year, whereas in summer visits 6.6% had hypertension at baseline and 6.1% at one year.

Vitamin B12 deficiency (<148 pmol/L) [33] was found in 2.3% of participants at baseline which decreased to 0.3% at follow-up. Optimal vitamin B12 (>450 pmol/L) [34] was seen in 39% of participants at baseline which increased to 82% at follow-up.

There was a significant negative association found between blood pressure and serum 25(OH)D level at baseline (systolic BP: Standardized Coefficient −0.07, *p* < 0.001; diastolic BP: Standardized Coefficient = −0.1, *p* < 0.001). After correcting for age, sex, BMI, AA: EPA and ethnicity, the association with 25(OH)D remained significant for diastolic BP (systolic BP: Standardized Coefficient = −0.03, *p* = 0.07, diastolic BP: Standardized Coefficient = −0.05, *p* = 0.001).

Baseline prevalence of systolic and diastolic hypertension was 19.4% and 11.4%, respectively, with hypertension (systolic and diastolic) in 7.3% (592 participants out of 8155). Pre-hypertension, with systolic BP in the range of 121–139 mmHg and diastolic BP of 81–89 mmHg, was seen in 52.6% of participants. Out of 592 participants (7.3%) who were hypertensive at baseline, 85 (14.4%) were vitamin D deficient, and 286 (48.3%) were vitamin D insufficient. At baseline, serum 25(OH)D levels were significantly lower in hypertensive participants (81 ± 35 nmol/L) compared with normotensives (87 ± 36 nmol/L) (*p*-value = 0.001).

Because our sample of participants represented a wide range of ages, 18–96 years old, we compared BP measures at baseline with respect to their age (Table S1). Mean systolic BP, pulse pressure and MAP increased with age. The number of hypertensive cases increased with age category as follows: <35 years: 11 (1.9%), 35–44 years: 30 (5.1%), 45–64 years: 336 (56.7%), and ≥65 years: 215 (36.3%). As such, age was controlled for in the further analysis.

### 3.2. Longitudinal Analysis

After an average of one year in the program, the prevalence of vitamin D deficiency significantly decreased to 2% (down from 11% at baseline) and insufficiency decreased to 14% (down from 42% at baseline). Thirty three per cent of participants at one year were taking vitamin D supplements at doses above 8000 IU/day. Sixty one percent of participants achieved the target vitamin D status, a serum 25(OH)D concentration above 100 nmol/L (<250 nmol/L), compared to 27% at baseline. There were no cases of toxicity [35].

We compared measures at baseline to one year in whole population (Table 2). We found a significant increase in mean serum 25(OH)D (87 ± 37 to 113 ± 39 nmol/L) and mean blood pressure parameters remained unchanged (systolic BP 125 ± 17 to 125 ± 17 mmHg (*p* = 0.10); diastolic BP 77 ± 10 to 77 ± 9 mmHg (*p* = 0.10)). Of those hypertensive at baseline (*n* = 592), 71.1% (*n* = 421) were no longer in the hypertensive range at follow-up. Among those with hypertension, 44% and 49% were on BP-lowering medication at baseline and follow-up, respectively. There was no significant difference in the reduction of systolic blood pressure (−12.7 ± 20.6 mmHg vs. −12.6 ± 18.2, *p* = 0.7) and diastolic blood pressure (−11.2 ± 10.8 mmHg vs. −9.7 ± 9.6, *p* = 0.07) between participants who did and did not use BP-lowering medication. Over 9% of participants who were hypertensive and on BP-lowering medication at program entry discontinued use during the year in the program.

Of hypertensive participants at baseline who were not taking any BP-lowering medications throughout the year in the program, 73% had reduced blood pressure at follow-up with 7% in the normotensive range and 66% in the pre-hypertensive range (Chi-Square, *p* < 0.001). In those who were normotensive at baseline 1.1% had hypertension at one year (Table S2).

We compared changes in blood pressure measures among normotensive, pre-hypertensive, and hypertensive patients in both the whole population and after excluding participants on BP-lowering medications at any time after joining the program (Table S3). In both the whole population and in the sample of participants not taking BP-lowering medications, there was a significant reduction in BP measures in the pre-hypertensive and hypertensive participants. 

As a final comparison, we assessed blood pressure changes among hypertensive participants whose observations were made either during the winter or summer season (Table S4). Following a mean serum 25(OH)D level increase of 30 nmol/L in both groups, in comparison with winter observations, those with summer observations had a greater reduction in systolic BP (−10.0 ± 17.4 vs. −13.5 ± 20.1 mmHg, respectively) and PP (−0.22 ± 14.9 vs. −3.1 ± 17.6 mmHg, respectively). At program entry, 14% of those observed during the winter were vitamin D deficient compared to 8% in summer; after a year of vitamin D supplementation only 2% of individuals in either group were vitamin D deficient.

### 3.3. Nested Case-Control Study

**Figure 1:**
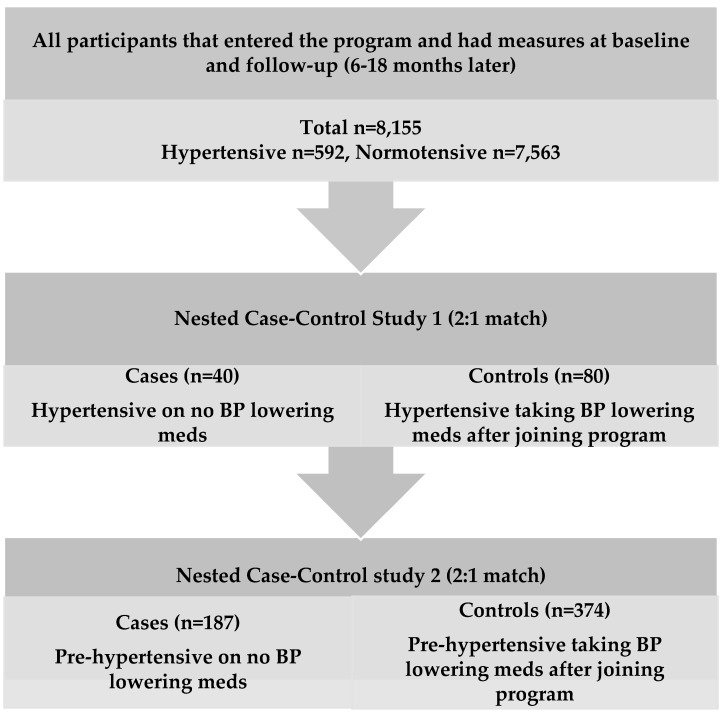
Flowchart of Database Analyses Approach.

To first investigate the effect of vitamin D supplementation, taken in combination with other nutrients, on blood pressure status in participants who were hypertensive, we selected hypertensive individuals not taking BP-lowering medication (*n* = 40) (Table 3). We compared these cases to a control group (i.e., hypertensive individuals who initiated BP-lowering medications after joining the program (*n* = 80)) that were matched based on age, sex and BMI. Next, to determine the effect of vitamin D supplementation on participants who were in the pre-hypertensive range (systolic BP 121–140 mmHg and diastolic BP 81–90 mmHg) and not taking BP-lowering medication (cases, *n* = 187), we compared this group to matched controls who initiated BP-lowering medications after joining the program (controls, *n* = 374) (Figure 1).

There were no major health concerns reported by either cases or controls with the exception of elevated blood pressure among both groups. Self-reported general health, fruits and vegetables consumption, and drinking status were not significantly different between groups (data not shown). In terms of ethnicity, a higher percentage of cases were Asian (10.8% vs. 7.6%) compared to controls. Fewer cases currently used tobacco products in comparison with controls (3.0% vs. 22.4%) and more were active (57.1% vs. 33.3% with moderate physical activity) compared to control group. These parameters were considered as confounders in further analysis. Cases and controls were otherwise comparable in terms of baseline systolic and diastolic blood pressure, pulse pressure and MAP, vitamin D supplementation dose and markers of nutritional status (serum 25(OH)D concentration, serum vitamin B12 level, and AA:EPA ratio) (Table 3). None of the cases reported initiation of BP-lowering medication after joining the program.

Nested case-control study 1: Both cases (hypertensive and not taking BP-lowering medication) and controls (hypertensive with the initiation of BP-lowering medication after joining the program) experienced significant reductions in systolic and diastolic blood pressure and MAP over one year (Table 3). There were no significant differences in blood pressure, systolic or diastolic, pulse pressure, MAP or nutritional markers changes between case and control groups (Table 3).

Nested case-control study 2: There was a slight but significant reduction in systolic and diastolic blood pressure and MAP in both case and control groups. The changes in vitamin D supplementation dose, serum 25(OH)D, serum vitamin B12 and AA: EPA over time were not significantly different between cases and controls (Table 3).

Binary logistic regression was utilized to determine if increased serum 25(OH)D was an independent predictor for blood pressure indices (Table 4). Although we were principally interested in whether achieving vitamin D sufficiency influenced the outcome, we also considered several other factors that might affect blood pressure measures including lifestyle changes (tobacco use, physical activity level, fruit, and vegetable consumption) and BP-lowering medication use. As such, we examined vitamin D status changes among categories of insufficiency and “optimal” (>100 nmol/L). After correcting for probable confounding factors including age, sex, season, ethnicity, BMI status, AA: EPA, CRP, vitamin B12, tobacco use, fruit and vegetable consumption, physical activity, and BP-lowering medications, only participants that were vitamin D insufficient at baseline that achieved optimal serum 25(OH)D status at follow-up (≥100 nmol/L) had a lower risk of hypertension (OR = 0.10, 95% CI: 0.01, 0.87, *p* = 0.03). Compared to other ethnicities, being Asian or North American was significantly associated with lower incidence of hypertension. Consumption of five and more servings of fruit and vegetable per day was associated with a decreased risk of hypertension (OR = 0.28 95% CI, 0.07, 1.1, *p* = 0.07).

## 4. Discussion

We found a significant independent association between low serum 25(OH)D levels and higher systolic and diastolic blood pressure. Overall, having insufficient vitamin D levels at baseline and achieving optimal 25(OH)D levels (≥100 nmol/L) conferred a significant risk reduction for hypertension at one year. We found that there was no significant difference in the BP lowering effect between hypertensive or pre-hypertensive participants on vitamin D supplementation and those strictly on BP-lowering medication. Together, the results suggest that 25(OH)D concentrations above 100 nmol/L may reduce the risk of hypertension and, in fact, may help reduce blood pressure in hypertensive and pre-hypertensive individuals. To achieve and maintain a serum 25(OH)D concentration of 100 nmol/L, at least 4000 IU/day (100 µg/day) of vitamin D is required [26].

In addition to the present study, others have shown vitamin D supplementation provides a blood pressure lowering effect in specific subpopulations, including patients with hypertension, low serum vitamin D status or cardiometabolic diseases in other studies [36,37,38,39,40,41,42,43]. A recent randomized controlled trial sub-study found lower blood pressure in vitamin D deficient individuals when treated with high-dose vitamin D supplementation long-term to maintain serum 25(OH)D above 100 nmol/L [43]. Our findings, however, are not consistent with all the published literature [44,45,46], although it is worth noting that the vast majority of these studies have included normotensive individuals in which we would not expect a reduction, and/or populations with vitamin D sufficiency at baseline. Further, null results have been found when supplementing with insufficient doses of vitamin D to achieve “optimal” serum 25(OH)D concentrations [9,47,48,49,50]. The present study would suggest that a physiological level, a serum 25(OH)D concentration above 100 nmol/L, is optimal.

Vitamin D supplementation doses differed between individuals because of the focus on serum 25(OH)D concentration—the program objective is to achieve physiological 25(OH)D levels above 100 nmol/L. There is a known, large inter-individual variation in dose–response to vitamin D that is also dependent on BMI, such that overweight and obese individuals require 2–3 times the dose of vitamin D that an individual with a normal BMI would require [51]. The effects found in hypertensive individuals cannot be attributed to vitamin D alone. However, only a reduced risk for hypertension was found when individuals who were vitamin D insufficient had achieved 25(OH)D levels above 100 nmol/L at follow-up. This suggests that the modest effect of vitamin D supplementation may require a year or more to be recognized. In support, the other two nutrient supplements for which we had serum measures, vitamin B12 and omega 6:3 ratios, did not have a significant contribution to blood pressure changes. Heaney outlined a set of criteria for investigating the effects of nutrients using the evidence-based medicine model which included ensuring that the nutrient status of the population is similar [29]. To ensure that the effect of only one nutrient is tested the co-nutrient status of the population must be optimal. It may be that all the nutrients supplied in the program were necessary to achieve optimal vitamin and mineral status in order for vitamin D to have a detectable impact on blood pressure. As mentioned, we are not able to rule out the influence of the other supplements. It would have been ideal if the other nutrients were introduced for a time before vitamin D supplementation was started, but this database analysis is limited by the realities of the program offered. Every biochemical pathway in the human body relies on multiple vitamins and minerals as cofactors to various steps [52]. The optimization of one nutrient such as vitamin D cannot correct a deficiency of other nutrients; it can only ensure that the various tasks that involve vitamin D are not compromised. However, nearly every cell requires vitamin D for proper function [53]. Vitamin D status may be the most relevant nutritional indicator of overall health.

If we are to consider Hill’s criteria, which are temporality, strength, dose–response relationship, consistency, plausibility, analogy, specificity and coherence, the evidence of a role for vitamin D in preventing or attenuating hypertension is moderate to good. Biological plausibility is provided through several mechanisms, including its immunomodulatory and anti-inflammatory effects. Vitamin D may regulate blood pressure through its actions on the renin-angiotensin activity, as an endogenous inhibitor leading to a decrease in blood pressure [12,54]. Vitamin D may also act to improve endothelial function [9] and attenuate the effect of parathyroid hormone [11]. There is strength of association and in the current analyses the effect size for blood pressure is significant (80% for systolic, 105% for diastolic and 107% for MAP). A dose–response relationship is suggested with increasing serum 25(OH)D from the deficient range to the physiological range (>100 nmol/L) and associated with significant reductions in systolic (−7.5 mmHg), diastolic (−4.4 mmHg) and MAP (−5.3 mmHg). As discussed above, the results of the present study are consistent with others investigating benefit of vitamin D supplementation for hypertension [7,20,41,43,55,56,57] and provide support for the reasoning for discrepant results [46]. Specificity and temporality are suggested by observational data and the current study lends support, but definitive evidence is still lacking. Coherence may be considered established given that negative studies with design flaws are rebutted by positive studies that account for design deficiencies.

The relationship between blood pressure and serum 25(OH)D level would likely remain similar across different seasons if other factors that affect blood pressure level during the winter months are taken into account, including acute response to environmental temperature, increases in sympathetic tone, noradrenaline concentrations, sodium uptake and body mass index [58,59]. In the current study, vitamin D supplementation aimed to achieve an optimal level of serum 25(OH)D and BP measures decreased in both the winter and summer seasons. However, we did observe a greater reduction in systolic BP during the summer than in the winter months, likely as a result of some of the other probable confounding factors affecting BP during the winter.

The current investigation has strength in its large sample size. We utilized a convenient sample of participants in a prevention program, with more than 8000 participants undergoing a clinical intervention. We also considered other factors that might influence the effect of vitamin D supplementation on blood pressure. There are several limitations to the present analysis. Data were not available to distinguish essential hypertension from secondary hypertension, which is mostly related to hyperaldosteronism, and vitamin D supplementation might closely influence the renin, angiotensin, and aldosterone pathway in the body [60]. Complete information on blood pressure medication dose and compliance was not available. The results are not applicable to an optimal dose of vitamin D as supplementation levels differed among participants (supplement recommendations in the range of 1000 to 20,000 IU/day), but the target was to achieve a 25(OH)D concentration above 100 nmol/L and differences were necessary based on individual requirements. The compliance of the population is considered a limiting factor and the possibility of a Hawthorne effect in these participants should be noted. In these analyses, participants were included who had both baseline and follow-up measures recorded, thus a selection bias is a probability. However, when these analyses were repeated using an intent-to-treat protocol, the results did not differ (data not shown).

## 5. Conclusions

The present analysis showed that achieving 25(OH)D concentrations ≥100 nmol/L in a hypertensive population was associated with a significant decrease in systolic and diastolic blood pressure and mean arterial pressure. To achieve and maintain a serum 25(OH)D concentration of 100 nmol/L, at least 4000 IU/day (100 µg/day) of vitamin D is required. In addition to advocating lifestyle modification and blood pressure lowering medication prescription, vitamin D supplementation may offer a simple, safe and cost-effective method for reducing blood pressure in vitamin D insufficient and hypertensive individuals.

## Figures and Tables

**Table 1:** Baseline Demographics and mean values within categories of vitamin D status.

Parameter at Baseline	All	Serum 25(OH)D					
		0–50	50–100	100–150	150–200	200–250	≥250
Number	8155	867	5059	1809	306	69	45
25(OH)D, nmol/L	87 ± 37	39 ± 8 ^f^	75 ± 13 ^e^	118 ± 13 ^d^	169 ± 14 ^c^	220 ± 16 ^b^	292 ± 38 ^a^
Vitamin D supplement dose, IU/day	1600 ± 2500	380 ± 1300 ^f^	1200 ± 2000 ^d^	2600 ± 2800 ^c^	4100 ± 3800 ^b^	5400 ± 4200 ^a^	6300 ± 500 ^a^
Vitamin B12, pmol/L	470 ± 310	320 ± 170 ^e^	440 ± 280 ^d^	580 ± 370 ^c^	680 ± 380 ^bc^	820 ± 500 ^a^	720 ± 350 ^b^
AA/EPA ratio	16 ± 8	21 ± 7 ^a^	17 ± 8 ^b^	14 ± 8 ^c^	12 ± 7 ^cd^	11 ± 7 ^d^	12 ± 10 ^cd^
Systolic BP, mmHg	125 ± 17	126 ± 16 ^a^	126 ± 17 ^a^	124 ± 17 ^ab^	123 ± 17 ^ab^	119 ± 18 ^b^	118 ± 14 ^b^
Diastolic BP, mmHg	77 ± 10	79 ± 10 ^a^	77 ± 10 ^ab^	76 ± 10 ^ab^	76 ± 10 ^ab^	75 ± 10 ^b^	75 ± 9 ^b^
Pulse Pressure, mmHg	48 ± 13	47 ± 12 ^ab^	48 ± 13 ^a^	48 ± 13 ^a^	47 ± 14 ^ab^	44 ± 14 ^b^	44 ± 9 ^b^
MAP, mmHg	93.2 ± 10.9	94.6 ± 10.9 ^a^	93.5 ± 10.9 ^a^	92.0 ± 10.8 ^ab^	91.6 ± 11.0 ^ab^	89.3 ± 11.4 ^b^	89.3 ± 9.6 ^b^
Age, year	56 ± 15	51 ± 15 ^c^	57 ± 15 ^a^	57 ± 15 ^a^	56 ± 15 ^ab^	53 ± 17 ^b^	52 ± 14 ^bc^
BMI, kg/m^2^	27 ± 6	30 ± 6 ^a^	28 ± 6 ^b^	26 ± 5 ^bc^	25 ± 5 ^cd^	24 ± 4 ^d^	24 ± 4 ^d^
WC, cm	93 ± 15	100 ± 16 ^a^	94 ± 15 ^b^	88 ± 13 ^c^	86 ± 14 ^cd^	84 ± 12 ^cd^	81 ± 11 ^d^
Female, *n* (%)	4890 (60)	403 (46)	2968 (59)	1229 (68)	209 (68)	48 (70)	33 (73)
Ethnicity, *n* (%)	7640	788	4756	1704	287	64	41
North American	5782 (76)	478 (61)	3562 (75)	1409 (83)	243 (85)	57 (89)	33 (80)
Asian	776 (10)	109 (14)	540 (11)	107 (6)	15 (5)	3 (5)	3 (7)
Aboriginal	304 (4)	112 (14)	159 (4)	30 (2)	3 (1)	0	0
European	492 (6.4)	29 (4)	300 (6)	129 (7)	26 (9)	4 (6)	4 (10)
Others	286 (3.7)	60 (7)	195 (4)	29 (2)	1 (0)	0	1 (3)
BMI status, *n* (%)	8155	867	5059	1809	306	69	45
Underweight	101 (1.2)	7 (1)	48 (1)	32 (2)	6 (2)	6 (9)	2 (4)
Normal weigh	2927 (36)	203 (23.2)	1693 (33.5)	815 (45)	159 (52)	31 (44)	26 (58)
Overweight	2970 (36.4)	293 (33.8)	1884 (37)	647 (36)	105 (34)	26 (38)	15 (33)
Obese	2157 (26.4)	364 (42)	1434 (28.5)	315 (17)	36 (12)	6 (9)	2 (5)
Self-reported HTN, *n* (%)	1378 (27.5)	119 (26.5)	939 (29)	281 (25)	30 (17)	7 (17)	2 (8)
Male: Female (if hypertensive)	1.25:1	2.69:1	1.32:1	0.60:1	1.83:1	1.50:1	0
History of cardiac disease, *n* (%)	381 (7.6)	32 (7)	242 (7.5)	93 (8.4)	12 (7)	1 (2.4)	1 (2.4)
BP lowering medication, *n* (%)	1291 (32)	94 (31)	906 (34)	255 (29)	27 (20)	7 (23)	2 (13)
Season, *n* (%)							
Winter (November–April)	3836 (47)	523 (60)	2162 (43)	900 (50)	181 (59)	42 (61)	28 (62)
Summer (May–October)	4319 (53)	344 (40)	2897 (57)	909 (50)	125 (41)	27 (39)	17 (38)

Different letters represent Tukey’s test, post-hoc One-Way ANOVA; 25(OH)D = 25 hydroxyvitamin D; AA = Arachidonic acid; BP = blood pressure; EPA = Eicosapentaenoic Acid; MAP = Mean Arterial Pressure; BMI = Body Mass Index; WC = waist circumference; HTN = hypertension.

**Table 2:** Comparison of measures over one year in the whole population.

Parameter	*n*	Baseline Mean ± SD	One-Year Mean ± SD	*p*-Value *
BMI (kg/m^2^)	8155	27 ± 6	28 ± 6	<0.001
Serum 25(OH)D (nmol/L)	8155	87 ± 37	113 ± 39	<0.001
Vitamin D supplementation dose (IU/day)	8127	1600 ± 2500	5200 ± 4300	<0.001
Vitamin B12 (pmol/L)	7966	470 ± 310	1200 ± 1100	<0.001
Systolic BP (mmHg)	8155	125 ± 17	125 ± 17	0.10
Diastolic BP (mmHg)	8155	77 ± 10	77 ± 9	0.10
Pulse Pressure (mmHg)	8155	48 ± 13	48 ± 14	0.09
MAP (mmHg)	8155	93 ± 11	93 ± 11	0.10
AA:EPA	1821	16 ± 8	11 ± 7	<0.001

* Paired T-test; BP = blood pressure; MAP = Mean Arterial Pressure; BMI = body mass index; AA = Arachidonic acid; EPA = Eicosapentaenoic Acid.

**Table 3:** Comparison of hypertensive cases on no BP-lowering medications with hypertensive controls on BP-lowering medications after entry to program (age-, sex- and BMI-matched).

	Cases (*n* = 40) Hypertensive on No BP Meds		Controls (*n* = 80) Hypertensive on BP Meds after Joining Program			Cases (*n* = 187) Pre-Hypertensive on No BP Meds		Controls (*n* = 374) Pre-Hypertensive on BP Meds after Joining Program		
Parameter	Mean ± SD	95% CI	Mean ± SD	95% CI	*p* Value ^a^	Mean ± SD	95% CI	Mean ± SD	95% CI	*p* Value ^a^
**Systolic blood pressure (mmHg)**										
Baseline	156 ± 15	151 to 161	155 ± 16	151 to 158	0.61	133 ± 10	131 to 134	136 ± 12	134 to 137	0.01
Follow-up	138 ± 21 *	131 to 145	141 ± 17 *	137 to 145	0.40	130 ± 15 *	128 to 132	135 ± 17	133 to 137	<0.01
Changes	−18 ± 19	−24 to −12	−14 ± 21	−18 to −9	0.25	−3 ± 16	−5 to −0.28	−1 ± 19	−1 to 1.3	0.23
**Diastolic blood pressure (mmHg)**										
Baseline	96 ± 8	94 to 99	96 ± 7	94 to 97	0.92	81 ± 7	80 to 82	81 ± 8	80 to 82	0.59
Follow-up	84 ± 12 *	80 to 87	84 ± 11 *	81 to 86	0.97	79 ± 10 *	78 to 80	79 ± 10 *	78 to 80	0.74
Changes	−12 ± 12	−16 to −9	−12 ± 12	−15 to −10	0.97	−2 ± 8	−4 to −1.3	−2 ± 10	−3 to −0.85	0.46
**Pulse Pressure (mmHg)**										
Baseline	60 ± 12	60 to 64	59 ± 13	56 to 62	0.56	52 ± 12	50 to 53	55 ± 14	53 to 56	0.01
Follow-up	55 ± 16 *	49 to 60	58 ± 13	55 to 61	0.27	52 ± 13	50 to 54	56 ± 16	54 to 58	<0.01
Changes	−5 ± 17	−11 to −0.44	−1 ± 17	−5 to 2	0.17	0 ± 15	−2 to 2	1 ± 17	−0.5 to 3	0.39
**Mean Arterial Pressure (mmHg)**										
Baseline	116 ± 10	113 to 119	116 ± 9	114 to 118	0.73	98 ± 6	98 to 99	99 ± 6	98 to 100	0.24
Follow-up	102 ± 14 *	97 to 106	103 ± 12 *	100 to 105	0.69	96 ± 8 *	95 to 97	98 ± 10 *	97 to 99	0.05
Changes	−14 ± 12	−18 to −11	−13 ± 13	−16 to −10	0.53	−2 ± 9	−4 to −1	−1 ± 11	−3 to −0.34	0.26
**Serum 25(OH)D (nmol/L)**										
Baseline	82 ± 38	70 to 95	83 ± 42	73 to 92	0.93	83 ± 32	79 to 88	87 ± 36	83 to 91	0.24
Follow-up	113 ± 35 *	102 to 124	110 ± 37 *	102 to 119	0.67	113 ± 36 *	108 to 118	112 ± 34 *	108 to 115	0.67
Changes	31 ± 35	20 to 43	27 ± 43	18 to 37	0.64	30 ± 36	24 to 35	25 ± 38	21 to 29	0.15
**Vitamin D supplementation dose**										
Baseline	1900 ± 2800	970 to 2800	2000 ± 2800	1300 to 2600	0.87	1400 ± 2000	1200 to 1700	1700 ± 2500	1500 to 2000	0.17
Follow-up	5900 ± 4600 *	4400 to 7300	6600 ± 4900 *	5500 to 7700	0.42	5500 ± 4100 *	5000 to 6100	5600 ± 4200 *	5100 to 6000	0.88
Changes	4000 ± 5000	2400 to 5600	4700 ± 5500	3400 to 6000	0.52	4100 ± 4400	3500 to 4700	3800 ± 4700	3400 to 4300	0.43
**Vitamin B12 (pmol/L)**										
Baseline	460 ± 310	360 to 560	440 ± 260	380 to 500	0.63	440 ± 270	400 to 480	520 ± 370	480 to 560	0.01
Follow-up	1500 ± 1300 *	1000 to 1900	1300 ± 960 *	1100 to 1500	0.45	1300 ± 1000 *	1100 to 1400	1400 ± 1300 *	1200 to 1500	0.38
Changes	1040 ± 1300	580 to 1500	860 ± 960	670 to 1100	0.55	860 ± 1900	680 to 970	880 ± 1300	720 to 980	0.30
**AA:EPA ratio ^#^**										
Baseline	15 ± 6.4	10 to 19	15 ± 7.5	5.6 to 18	0.93	16 ± 7.1	12 to 16	15 ± 7.1	12 to 19	0.15
Follow-up	6.1 ± 2.8 *	2.8 to 9.2	7.9 ± 4.3 *	4.1 to 10	0.37	8.6 ± 4.4 *	7.0 to 10	9.7 ± 6.9	7.9 to 11 *	0.39
Changes	−8.7 ± 4.7	−14 to −3.7	−6.8 ± 5.7	−11 to 0.89	0.26	−7.2 ± 5.1	−7.4 to −3.5	−4.9 ± 7.7	−10 to −5.5	0.17

CI = Confidence Interval; ^a^ Independent-Samples Student T-test; * Denotes a significant difference between baseline and one year; Paired T Test; *p*-value <0.05. BP = blood pressure; MAP = Mean Arterial Pressure; BMI = body mass index; AA = Arachidonic acid; EPA = Eicosapentaenoic Acid; ^#^ AA:EPA ratio was available for *n* = 21 cases and *n* = 42 controls.

**Table 4:** Predictors of blood pressure using binary logistic regression modeling.

Dependent Variable	Model	Co-Variables	Exp (B)	*p*-Value	95% CI
Multivariate analysis					
HTN (R^2^ = 0.03, *p* < 0.001)	1	Age	1.02 *	<0.001	1.01–1.03
		Sex (male)	1.91 *	<0.01	1.50–2.43
		25(OH)D (BL < 50 and FL ≥ 100)	Ref	0.07	
		25(OH)D (BL 50–100 and FL ≥ 100)	0.64 *	0.04	0.41–0.99
		25(OH)D (BL ≥ 100 and FL ≥ 100)	0.58 *	0.02	0.36–0.92
HTN (R^2^ = 0.27, *p* < 0.001)	2	Age	1.03	0.34	0.97–1.1
		Sex (male)	3.5	0.11	0.73–17
		25(OH)D (BL < 50 and FL ≥ 100)	Ref	0.08	
		25(OH)D (BL 50–100 and FL ≥ 100)	0.10 *	0.03	0.01–0.87
		25(OH)D (BL ≥ 100 and FL ≥ 100)	0.46	0.43	0.07–3.2
		Season (Winter)	1.0	0.98	0.27–3.7
		Vitamin B12 (BL < 450 and FL ≥ 450)	1.2	0.76	0.30–5.1
		Ethnicity (other ethnics)	Ref	0.12	
		North American	0.45 *	0.03	0.21–0.96
		Asian	0.25 *	0.01	0.08–0.78
		European	0.44	0.15	0.17–1.5
		Aboriginal	0.53	0.23	0.19–1.5
		BMI (Normal weight)	Ref	0.11	
		Overweight	0.67	0.65	0.12–3.8
		Obese	3.6	0.11	0.75–17
		AA:EPA ratio	0.88	0.24	0.71–1.1
		Inflammation (hs-CRP)	0.85	0.62	0.41–1.7
		Fruit and vegetable consumption (≥5 servings/day)	0.28	0.07	0.07–1.1
		Physical activity (none)	Ref	0.95	
		Mild	1.8	0.64	0.15–23
		Moderate	1.3	0.82	0.11–16
		Strenuous	1.6	0.77	0.06–45
		Tobacco use (yes)	2.1	0.40	0.36–13
		BP lowering medication (yes)	0.20	0.21	0.02–2.4

Binary Logistic Regression, * indicates *p* value <0.05; HTN = hypertension; BL = baseline; FL = follow-up; AA = Arachidonic Acid; EPA = Eicosapentaenoic Acid; BMI = body mass index; BP = blood pressure; hs-CRP = high sensitivity C-reactive protein; Reference group for serum vitamin B12 (BL ≥ 450 and FL ≥ 450 pmol/L).

## Supplementary Materials

The following are available online at www.mdpi.com/2072-6643/9/11/1244/s1. Table S1: Baseline comparison of blood pressure measures, serum 25(OH)D and vitamin D supplementation dose according to age groups, Table S2: Comparison of blood pressure status between baseline and one year (Participants who did not take BP-lowering medication at baseline and follow-up), Table S3: Blood pressure measures changes over time in whole population and after excluding participants on BP-lowering medication after joining the program, according to BP status, Table S4: Comparison of changes in blood pressure measures in hypertensive participants according to the season of observation.
