# Supplementary material for: The Association between Serum 25(OH)D Status and Blood Pressure in Participants of a Community-Based Program Taking Vitamin D Supplements
Source: Nutrients. 2017 Nov 14;9(11):1244. doi: 10.3390/nu9111244 (PMC5707716; doi:10.3390/nu9111244)
Supplement: Supplementary file 1 [file nutrients-09-01244-s001.pdf]

Supplementary Material

Table S1. Baseline comparison of blood pressure measures, serum 25(OH)D and vitamin D supplementation dose according to age groups

Baseline measure	Age Range (yr)	N	Mean	SD	P-value*
**Systolic blood pressure (mmHg)**	< 35	947	116[d]	12	< 0.001
	35-44	831	118[c]	14	
	45-64	3,823	125[b]	16	
	≥ 65	2,554	132[a]	18	
	Total	8,155	125	17	
**Diastolic blood pressure (mmHg)**	< 35	947	73[d]	9	< 0.001
	35-44	831	76[c]	10	
	45-64	3,823	78[a]	10	
	≥ 65	2,554	77[b]	10	
	Total	8,155	77	10	
**Pulse Pressure (mmHg)**	< 35	947	42[c]	9	< 0.001
	35-44	831	43[c]	10	
	45-64	3,823	46[b]	12	
	≥ 65	2,554	54[a]	15	
	Total	8,155	48	13	
**Mean Arterial Pressure (mmHg)**	< 35	947	88[d]	10	< 0.001
	35-44	831	90[c]	11	
	45-64	3,823	94[b]	11	
	≥ 65	2,554	95[a]	11	
	Total	8,155	93	11	
**Serum 25(OH)D (nmol/L)**	< 35	947	84[bc]	41	< 0.001
	35-44	831	83[c]	39	
	45-64	3,823	87[ab]	39	
	≥ 65	2,554	89[a]	31	
	Total	8,155	87	37	
**Vitamin D dose (IU/d)**	< 35	947	1400[b]	2800	< 0.001
	35-44	831	1500[ab]	2700	
	45-64	3,823	1700[a]	2600	
	≥ 65	2,554	1500[ab]	2000	
	Total	8,155	1600	2500	
**BMI (kg/m^2)**	< 35	947	26[c]	5	< 0.001
	35-44	831	27[b]	6	
	45-64	3,823	28[a]	6	
	≥ 65	2,554	28[a]	5	
	Total	8,155	27	6	

*One-Way ANOVA, different superscripts denote differences using post-hoc Tukey’s test

Table S2. Comparison of blood pressure status between baseline and one year (Participants who did not take BP-lowering medication at baseline and follow-up)

	Baseline Status	One Year Status		
		Normal	Pre-hypertension	Hypertension
**Whole population**	Normal	548 (65)	289 (34)	9 (1.1)
	Pre-hypertension	378 (36)	607 (59)	53 (5)
	Hypertension	8 (7)	77 (66)	32 (27)
**Age < 34 yr**	Normal	65 (77)	19 (23)	0
	Pre-hypertension	19 (51)	17 (46)	1 (3)
	Hypertension	0	1 (100)	0
**35 – 44 yr**	Normal	55 (81)	12 (18)	1 (1)
	Pre-hypertension	16 (42)	22 (58)	0
	Hypertension	0	4 (57)	3 (43)
**45 – 64 yr**	Normal	312 (68)	142 (31)	4 (1)
	Pre-hypertension	214 (41)	270 (52)	38 (7)
	Hypertension	3 (5)	39 (64)	19 (31)
**≥ 65 yr**	Normal	116 (49)	116 (49)	4 (2)
	Pre-hypertension	129 (29)	298 (68)	14 (3)
	Hypertension	5 (10)	33 (69)	10 (21)

Chi square test, p<0.001, Data presented as number (% within BP status at baseline)

Table S3. Blood pressure measures changes over time in whole population and after excluding participants on BP-lowering medication after joining the program, according to BP status

**Study population**		**Whole population (n=8,155)**				**After excluding participants on BP meds (n=4,251)**				**Between groups p value***
**Parameter**	**BP status**	**N**	**Mean**	**SD**	**P value**	**N**	**Mean**	**SD**	**P value#**	
Systolic blood pressure change	normotensive	3273	6.0[a]	14	< 0.01	1819	5.7[a]	14	< 0.001	0.58
	pre-hypertensive	4290	-3.8[b]	16		2156	-4.6[b]	16		0.81
	hypertensive	592	-12[c]	19		276	-13[c]	18		0.53
Diastolic blood pressure change	normotensive	3273	2.6[a]	9.6	< 0.01	1819	2.7[a]	10	< 0.001	0.93
	pre-hypertensive	4290	-2.0[b]	9.5		2156	-2.3[b]	9.4		0.72
	hypertensive	592	-10[c]	10		276	-10[c]	10		0.89
Pulse Pressure change	normotensive	3273	3.4[a]	12	< 0.01	1819	3.0[a]	12	< 0.001	0.48
	pre-hypertensive	4290	-1.9[b]	15		2156	-2.3[b]	15		0.61
	hypertensive	592	-1.6[b]	16		276	-3.0[b]	16		0.06
Mean Arterial Pressure change	normotensive	3273	3.8[a]	9.5	< 0.01	1819	3.7[a]	9.4	< 0.001	0.88
	pre-hypertensive	4290	-2.6[b]	9.6		2156	-3.1[b]	9.3		0.27
	hypertensive	592	-11[c]	11		276	-11[c]	11		0.98
Serum 25(OH)D change	normotensive	3273	24[b]	43	< 0.01	1819	23	42	0.01	0.97
	pre-hypertensive	4290	28[a]	40		2156	27	40		0.83
	hypertensive	592	31[a]	43		276	28	38		0.05

#One-way ANOVA, different letters show post-hoc test (Tukey test), *Independent T-test,

Table S4. Comparison of changes in blood pressure measures in hypertensive participants according to the season of observation

BP measure change	Season	N	Mean ± SD	P value
Systolic blood pressure (mmHg)	Winter	309	-10 ± 17	0.02
	Summer	283	-14 ± 20	
Diastolic blood pressure (mmHg)	Winter	309	-10 ± 10	0.47
	Summer	283	-11 ± 10	
Pulse Pressure (mmHg)	Winter	309	-0.22 ± 15	0.03
	Summer	283	-3.1 ± 18	
Mean Atrial Pressure (mmHg)	Winter	309	-10 ± 11	0.07
	Summer	283	-12 ± 12	
Serum 25(OH)D (nmol/L)	Winter	309	31 ± 46	0.81
	Summer	283	31 ± 39	

Independent-Samples T-test, winter=November-April, summer=May-October
